# Accelerating drug discovery for pregnant and lactating women living with HIV

**DOI:** 10.1002/jia2.25680

**Published:** 2021-03-04

**Authors:** Risa M Hoffman, Rosie Mngqibisa, Dawn Averitt, Judith S Currier

**Affiliations:** ^1^ Department of Medicine and Division of Infectious Diseases David Geffen School of Medicine at the University of California Los Angeles CA USA; ^2^ Durban International Clinical Research Site Enhancing Care Foundation Durban South Africa; ^3^ Women’s Research Initiative on HIV/AIDS (WRI)/The Well Project New York NY USA

**Keywords:** HIV, women, clinical trials, pregnancy, postpartum

On International Women’s Day, we honour the incredible advancements in science and public health that have resulted in improved health for the more than 1.5 million women living with HIV who become pregnant each year, as well as for their children and families [1]. These achievements have been possible through the participation of countless women in clinical trials and the dedication of researchers, programme planners and community members who have been at the forefront of ensuring women’s health has remained a priority on the global HIV research agenda. However, despite these important gains, the pace of drug discovery and approval for pregnant and lactating women remains unacceptably slow, with an approximate six‐year lag between antiretroviral licensure and any data in pregnancy [2]. Pregnant and lactating women are typically excluded from clinical trials in order to protect the mother and foetus from potential harm. However, excluding these women from trials instead shifts the risk of harm from occurring under trial settings in which informed consent and intensive monitoring are practiced, to occurring in routine care settings in which medications may be used despite a lack of data for evidence‐based management decisions. Therefore, the strategy of excluding pregnant and lactating women from clinical trials to avoid harm in fact only serves to increase risk for larger numbers of women who are exposed to medications with uncertain dosing, safety and efficacy data.

Advocacy for the women’s health research agenda has represented an important step towards future equity and includes leadership from the Task Force on Research Specific to Pregnant and Lactating Women (PRGLAC), sponsored by the National Institute of Child Health and Human Development (NICHD), and the Pregnancy and HIV/AIDS Seeking Equitable Study (PHASES) Working Group, sponsored by the National Institute of Allergy and Infectious Diseases (NIAID). These groups have outlined and are working to overcome barriers to research in pregnant and lactating women, which include liability concerns (legal, financial, reputational); lack of clarity on ethics; costs; a challenging regulatory environment; and a lack of timely preclinical reproductive toxicity and pharmacokinetic (PK) data [3, 4]. The PHASES Working Group has proposed a conceptual shift that includes defining pregnant women as a *complex population* rather than a *vulnerable population*; protecting women *through research* rather than *from research*; and striving for *fair inclusion* in research rather than *presumptive exclusion* [4]. Actualizing this frameshift will require significant adaptations in the approach to research in the following areas: (1) reducing regulatory barriers; (2) leveraging innovations in study design; and (3) strengthening infrastructure for research with an emphasis on global collaborations and multisectoral partnerships. Below, we further elaborate on each of these areas with a focus on tangible steps towards more equitable and timely inclusion of pregnant and lactating women in clinical research.

*Reducing regulatory barriers* is a critical step that can facilitate earlier enrolment of pregnant and lactating women in clinical trials to ensure PK, safety, and efficacy data for these populations are available when new drugs are approved. The majority of data for therapeutics in pregnancy arise from Phase IV studies; however, with more proactive planning, early preclinical reproductive toxicity data can allow for inclusion of pregnant women in Phase IIb/III studies. These studies can employ approaches to reduce risks, such as that used in the US Microbicide Trials Network’s DELIVER Study, in which women are enrolled in sequential cohorts, first in late pregnancy (36+ weeks) and, after safety is established for these women and their infants through interim data review, subsequent cohorts of women are enrolled in progressively earlier antepartum periods [5].

Additionally, requirements for women to stop study drug if they become pregnant during trial follow‐up slows the pace of discovery. In order to address concerns about risk and allow inclusion of these women in licensure trials, appropriate re‐consent language should be used, allowing women to weigh the risks and benefits of participation, assuming preclinical reproductive toxicity data do not suggest increased risk. Inclusion of women conceiving on study should involve the collection of PK data, extended follow‐up of all infants, reporting of pregnancy and infant outcomes using standardized definitions, and collecting information about background rates of adverse outcomes (including birth defects) in the trial countries where these women live to contextualize study outcomes.

*Leveraging innovations in study design* can improve the efficiency and timeliness of data availability. Complex Innovative Designs, including Master Adaptive Platform Trials, have been increasingly utilized in oncology research and most recently deployed for SARS‐ CoV‐2 in order to fast‐track drug discovery during the COVID‐19 pandemic. Adaptive trial design allows for pre‐specified modifications to the design based on interim data. Common adaptive designs use strategies such as sample size reassessment, early stopping for superiority or futility, response‐adaptive allocation (adapting the allocation ratio to favour enrolment in one arm of the study to improve the probability of selecting the best intervention), and seamless design (allowing for multiple integrated phases in one trial, such as collecting and using early pregnancy PK data to identify optimal drug dose for the next study phase) [6]. Master Adaptive Platform Trials use a master protocol and provide the ability to evaluate several interventions against a common control group, with adaptation rules for dropping ineffective interventions and introducing new arms. These trials can be perpetual and updated over time, and hold great promise for accelerating drug discovery and evidence‐based therapies for pregnant and lactating women.

*Strengthening infrastructure for research with an emphasis on global collaborations and multisectoral partnerships* will be necessary to carry forward the work defined above. This includes engagement of industry, regulatory agencies, academicians, government, non‐governmental organizations, existing clinical trial sites, and women from communities who have the highest stake in decisions about clinical trials. Drug discovery can be accelerated by implementing incentives for moving forward with early preclinical studies on reproductive toxicity and early PK studies in pregnancy, by requiring drug developers to assess their product in pregnant and lactating women during early drug development if these preclinical and PK data are favourable, and by setting standards for a minimum number of pregnant and lactating women to be included in these early studies. With collaborations between all stakeholders, early data can be leveraged to nimbly move to Phase III studies without prolonged delays between steps.

In conclusion, we propose, on this International Women’s Day, to move away from a passive approach to therapeutic discovery for pregnant and lactating women, and towards a proactive approach – one that will result in acceleration of knowledge and the optimization of health for large numbers of women and their children across the world. Pregnant and lactating women must be protected through research, not from research. We must achieve equitable and timely inclusion of all women in clinical trials.

## COMPETING INTERESTS

Risa M. Hoffman – Has worked as a consultant for GLG Member Solutions; Rosie Mngqibisa – None; Dawn Averitt – Consults for Merck; Judith S. Currier –none.

## AUTHORS’ CONTRIBUTIONS

All co‐authors worked together to develop the outline. Risa Hoffman wrote the first draft. Rosie Mngqibisa, Dawn Averitt, and Judith S. Currier provided edits. All authors have approved the final version.
